# Affective Outcomes of Group versus Lone Green Exercise Participation

**DOI:** 10.3390/ijerph17020624

**Published:** 2020-01-18

**Authors:** Mike Rogerson, Ian Colbeck, Rachel Bragg, Adekunle Dosumu, Murray Griffin

**Affiliations:** 1 School of Sport, Rehabilitation and Exercise Sciences, University of Essex, Colchester CO4 3SQ, UK; mgriffin@essex.ac.uk; 2 School of Life Sciences, University of Essex, Colchester CO4 3SQ, UK; colbi@essex.ac.uk (I.C.); aadosu@essex.ac.uk (A.D.); 3 Social Farms & Gardens, The GreenHouse, Hereford Street, Bristol BS3 4NA, UK; rachel@farmgarden.org.uk

**Keywords:** green exercise, group, social, mood, self-esteem, environment, nature

## Abstract

‘Green exercise’ (being physically active within a natural environment) research has examined the influence of environmental setting on health and wellbeing-related exercise outcomes. However, it is not known whether social exercise settings influence green exercise-associated changes in mood, self-esteem, and connection to nature. This study directly compared outcomes of participating in green exercise alone compared to in a group. Using repeated measures, counterbalanced and randomized-crossover design, participants (n = 40) completed two 3 km runs around sports fields. These fields had a relatively flat grass terrain, predominant view of trees, and open grassland. On one occasion participants ran alone and on the other they ran in a group of 4–5 participants. Questionnaire measures of mood, self-esteem, and connection to nature were completed immediately pre- and post-run. Across all of the measures, two-way mixed ANOVAs found that there were statistically significant effects for time but not for time-by-condition interactions. The simplest interpretation of this finding is that social setting does not influence individuals’ attainment of the psychological outcomes of green exercise participation. However, we discuss the possibility that more complex processes might underpin this finding.

## 1. Introduction

Since 2003, green exercise research has examined the influence of environmental setting on health and wellbeing-related exercise outcomes. In comparison with equivalent forms of exercise in indoor or built environments and in laboratories while viewing environmental scenes [1], exercise in greenspaces has been shown to increase levels of directed attention [2,3,4], improve mood [4,5,6], reduce levels of frustration and arousal, increase levels of meditation [7], and improve self-reported mental health across 8 weeks [8]. Positive expectancy can enhance some of these acute effects of the environment [9]. Two popular psychological outcomes reported by research focussing on the importance of environmental setting are mood and self-esteem [10,11,12,13,14]. This is in part due to their importance to adherence via affect–intention relationships [15,16] and the role of self-esteem in physical-activity behaviours via perceived self-efficacy and control [17,18,19,20,21,22].

A number of hypotheses, theories, and models have been proposed to explain the influences of the environment on exercise outcomes and the shaping of exercise behaviours [1]. While the biophilia hypothesis [23,24], stress-reduction theory [25], and attention-restoration theory [26] suggest the underpinnings of psychological responses to different types of environments (which researchers report to occur during exercise as well as at rest), the intertwining-pathways model of green exercise suggests that the reported outcomes of green exercise participation occur through two intertwined pathways comprising salutogenic effects of exposure to nature and behaviour-shaping effect of the environment [27]. More specifically, the ecological-dynamics perspective [28] describes that a dynamic interplay between the environment and individual continually reshape exercise experiences and thereby the associated outcomes, and that outcomes cyclically shape the interplay.

Resonating with the two latter theories, individuals’ level of psychological connection to nature can influence the affective outcomes of green exercise participation—individuals who feel more connected report greater positive changes from pre- to post-exercise [10]. The ecological-dynamics perspective suggests that this occurs because a greater level of connection enhances the affordance for and likelihood of individuals engaging in a psychologically restorative interaction with a greenspace environment. Although the construction of a connection with nature is thought to be relatively stable in the short term, it is shaped by experience. Therefore, an immersive bout of green exercise might function to increase the reported level of connection.

It is not known whether social exercise settings influence green exercise-associated changes in mood, self-esteem, or connection to nature. The majority of studies have examined psychological influences of exercise environments in individuals exercising alone [14,29,30,31], with a partner [32], in a group [33], or mass-participation events [10]. Studies on solo and group exercise have reported that greenspace environments enhance a range of psychological outcomes, such as increased positive and decreased negative mood/affect, directed attention, and enjoyment. This suggests that individual environment-level interactions associated with exercise seem likely to promote desirable psychological outcomes despite the varying impacts of social settings. However, the relative contributions of factors such as environmental and social settings to psychological outcomes are inter-related, transient, and dynamic. Psychological effects associated with social interactions during exercise might function to alter, detract from, or over-ride other pathways that are affected by exercise and environmental influence [27,32]. No studies have yet directly compared the outcomes of green exercise participation between the social settings of exercising alone and in a group. Indeed, across the exercise science literature, little research has been published that compares the affective outcomes of single acute bouts of exercising alone and in a group. Likely due to large individual differences in preferences, research has instead reported on explanations of individual preferences and the influence of social setting on motivation and adherence, often within prescribed exercise programmes.

There is merit to examining whether social setting influences the outcomes of single bouts of green exercise running. This may serve as a preliminary indication of how social settings might be optimised in addition to the environmental setting in order to enhance wellbeing-related parameters. 

This study examined the influence of group settings on some commonly reported psychological outcomes of green exercise participation. Although this study was considered to be exploratory, for the sake of analysing data using hypothesis-driven procedures the hypotheses for each variable (mood, self-esteem, and connection to nature) were as follows—(i) increases would occur in both conditions from pre- to post-exercise and (ii) increases would be greater when participants exercised alone than when they exercised in a group.

## 2. Materials and Methods 

### 2.1. Participants

Participants (n = 40; 20 males, 20 females) were university students and staff and members of the public from the local area, aged 21–68 years (mean age 36.43 ± 11.33 years). Participants were recruited through posters and electronic advertisements and were not paid for their participation. This study was approved by the University Ethics Committee. All participants were screened for exercise-associated risks using a Physical Activity Readiness Quesitonnaire (no participants were excluded) and gave informed consent for their participation.

### 2.2. Design and Procedure

The study design used a repeated-measures-counterbalanced and randomized-crossover design whereby participants completed two test occasions. On each occasion, participants completed a 3 km run. The only difference between conditions was that on one occasion they ran alone and on the other they ran in a group of 4–5 participants. On both test occasions participants completed composite questionnaires immediately pre- and post-run.

Each run was at the participant’s self-selected pace and comprised two 1.5 km laps of university sports fields. These had a relatively flat grass terrain, predominant views of trees and open grassland, and some views of buildings in the distance. There was an abundance of wildlife on the route, including squirrels and birds. In addition to a verbal description from a researcher before each run, route directions were positioned every 100 meters along the route to ensure route adherence. The entire route was visible to the researchers, both for safety and to ensure adherence. The route distance was chosen so that the exercise would have a likely duration of 10–20 min, which is sufficient for promoting positive changes in an adult’s mental state [34,35]. This duration was also popular in previous research comparing outdoor green exercise with built, urban, or simulated non-green exercise [3,6,7,14,29,30,32,33].

### 2.3. Measures

Age, sex, and data relating to the three measures of interest were collected via a composite questionnaire.

#### 2.3.1. Self-Esteem

The Rosenberg self-esteem scale is a validated and widely used ten-item measure of psychological wellbeing in physical-activity research [36,37,38]. Respondents indicated the extent to which they agreed with each of the ten statement items by ticking one of four boxes along a Likert scale from ‘strongly agree’ to ‘strongly disagree’. The associated scoring of each item was from 0 to 3 (creating an overall score of 0–30) [39]. Higher score values indicated better states of self-esteem.

#### 2.3.2. CNS

The connectedness to nature scale (CNS) is a “measure of individuals’ trait levels of feeling emotionally connected to the natural world” and consists of 14 items rated on a 5-point Likert scale ranging from 1 (strongly disagree) to 5 (strongly agree) [40]. To calculate the overall CNS score, item scores are summed (the total scale score ranges from 14 to 70) and then divided by 14 to give a minimum of 1 and a maximum of 5. Higher scores reflect a higher degree of affective connectedness to nature. A ‘state’ version (rather than trait) was created in order to assess the acute state of nature affiliation. This has been validated using undergraduate students and showed positive associations with environmental self-awareness, private self-awareness, ability to reflect, attentional capacity, and a negative association with public self-awareness [41]. The current study used a simplified version of the state-version CNS.

#### 2.3.3. Mood

The short version of the profile of mood states (POMS) [42,43] is comprised of six subscales—tension, depression, anger, vigour, fatigue, and confusion. Individuals completed the POMS by describing how they feel ‘right now’ via responses to 30 single-word mood-descriptor items along a five-point Likert-type scale. Each mood descriptor’s score ranges from 0 (‘not at all’) to 4 (‘extremely’). Raw scores were converted to ‘T scores’ as per McNair et al. [43]. Although individual subscales can be analysed alone, all subscales are interrelated and an overall mood score that accommodates this can be calculated by summing the negative subscale scores (tension, depression, anger, fatigue, and confusion) and subtracting the subscale score for vigour. This overall score is called total mood disturbance (TMD), with a greater score indicating a worse mood (minimum = 112; maximum = 282). Validity and reliability tests showed that the shortened version of the POMS is suitable for use in exercise contexts [44,45].

### 2.4. Statistical Analyses

For self-esteem and CNS, repeated-measures ANOVAs were conducted to assess the effects of time and possible time-by-condition interaction effects. A MANOVA was used for the POMS. An alpha level of 0.05 was used to indicate statistical significance. All analyses were conducted using SPSS version 25 (IBM Corp, Armonk, NY, USA).

## 3. Results

### 3.1. The Run

Mean temperature was very similar between group (11.4 ± 4.4 °C) and lone running (12.63 ± 2.70 °C) sessions. Across both conditions, the ambient temperature ranged from 6 °C (mostly cloudy) to 20 °C (mostly sunny).

Time taken to complete the 3 km was highly similar between conditions (group mean= 15.48 ± 2.70 min, in the range of 11.50–19.17 min; alone mean = 15.59 ± 2.38 min, in the range of 12.01–21.00 min).

### 3.2. Psychological Outcomes

For the CNS there was a large (η^2^ = 0.53 [46]) and statistically significant main effect on time (F_1,39_ = 43.48, *p* < 0.001) but not the time-by-condition interaction (F_1,39_ = 0.28, *p* = 0.60; η^2^ = 0.007).

For self-esteem there was a large (η^2^ = 0.45 [46]) and statistically significant main effect on time (F_1,39_ = 32.19, *p* < 0.001) but not the time-by-condition interaction (F_1,39_ = 0.45, *p* = 0.50; η^2^ = 0.012).

For the POMS, there was a large (η^2^ = 0.58) and statistically significant effect on time (F_6,34_ = 7.84, *p* < 0.001). There was a medium-sized (η^2^ = 0.124) but not statistically significant time-by-condition interaction effect (F_6,34_ = 0.81, *p* = 0.573).

Univariate analysis showed that for all of the POMS subscales there were large and statistically significant effects for time (tension F_1,39_ = 35.20, *p* < 0.001; η^2^ = 0.474; depression F_1,39_ = 9.92, *p* = 0.003; η^2^ = 0.020; anger F_1,39_ = 10.35, *p* = 0.003; η^2^ = 0.21; vigour F_1,39_ = 14.64, *p* < 0.001; η^2^ = 0.273; fatigue F_1,39_ = 1.275, *p* = 0.266; η^2^ = 0.032; confusion F_1,39_ = 16.91, *p* < 0.001; η^2^ = 0.302). Mean and standard deviation values for each measure at pre- and post-run in each condition are shown in Table 1.

## 4. Discussion

The purpose of this study was to provide a preliminary examination of the influence of group settings on some commonly reported psychological outcomes of green exercise participation. 

The hypotheses were that for each variable (mood, self-esteem, and connection to nature) (i) increases would occur in both conditions from pre- to post-exercise; and (ii) increases would be greater when participants exercised alone than when they exercised in a group.

Hypothesis (i) was supported across all variables, with large score improvements from pre- to post-exercise. For the measures of mood and self-esteem, this finding is consistent with previous green exercise research and literature [6,47,48,49]. Although beyond the scope of this study to examine, previous research has indicated many mechanisms underpinning the acute exercise-associated improvement in affect, such as the endorphin and monoamine hypotheses, secretion of other neurotransmitters, transient hypofrontality, distraction, and altered reactivity of the hypothalamic–pituitary–adrenal axis and other brain systems to stressors [50,51,52]. 

The CNS increase from pre- to post-exercise in both conditions is, to the authors’ knowledge, the first reporting of such a finding. It suggests that both group and lone green exercise participation could be employed as a means to increase public levels of nature connection. This is a key focus of conservation organisations given the links between connection to nature and pro-environmental attitudes and behaviours [53,54]. In addition to affective responses to exercise [15], enhanced connection to nature might also serve as motivation for future engagement in exercise behaviours.

Hypothesis (ii) was not supported—improvements in each measure were similar between solo and group exercise. The simplest interpretation of this finding is that social setting does not influence an individual’s achievement of the psychological outcomes of green exercise participation. However, there may be more complex processes underpinning this finding. In line with the proposition of dual-mode theory [55], the relative contributions of social and environmental settings may have differed between conditions. It might be that social influences of the group setting functioned to positively influence the outcome parameters while simultaneously reducing the occurrence or potency of individual (physical) environment-level interactions, which within the ‘alone’ condition functioned unimpeded to promote the reported psychological outcomes. The opposite interpretation is also possible—the positive effects of exercise and/or the environment were simply far larger than any influences of social setting. Although the design of the current study does not allow for conclusions about such environmental effects, previous research has frequently reported that greenspaces promote greater psychological improvements compared to exercise in built (real or simulated) or indoor environments [1,2,4,5,6,7,8,9,10,14,32]. If environmental settings do indeed influence psychological outcomes in this way, considering the affective improvements observed, the current findings suggest that practitioners should not fear possible lessening impacts of group settings on the cited benefits of selecting nature/greenspace environments for boosting psychological outcomes.

## 5. Limitations and Further Research

The current study builds on existing literature by using a robust design. However, further research should utilise a two-factor design to investigate how social and environmental settings may interact. Further research should also consider this research question in relation to other parameters previously reported within green exercise literature, such as directed attention, other affective states, and physiological and behavioural measures. A further limitation of this study was that data on participants’ preferences and usual behaviours regarding solo or group exercise were not collected. It is therefore not possible to know the extent to which individual differences in these factors were present and whether they may have been counterbalanced across the hypotheses and experimental design.

Although the current study did not find that social setting influenced the dependent variables, it is important to note that our sample consisted primarily of healthy young to middle-aged adults who reported relatively positive pre-run scores. Testing the current hypotheses in different cohorts, such as those experiencing depression, poor mood, or low self-esteem, is of great interest to scientific understanding and health practitioners. To this point, the current study could be developed through further research to also examine possible longer-term effects of social setting on outcomes and adherence to green exercise participation. For example, in relation to dual-mode theory, how does a group setting influence the phenomenological experience and psychological outcomes of green exercise when participation becomes a frequently repeated behaviour?

## 6. Conclusions

This study serves as a preliminary investigation of the possible importance of social setting in relation to previously reported affective outcomes of green exercise participation. No significant influence of social setting was found, offering a platform for debate over the interplay between environmental and social settings in relation to psychological experience and outcomes of exercise.

## Figures and Tables

**Table 1:** Mean ± standard deviation values for psychological measures.

	CNS		Self-Esteem		POMS TMD	
	Pre-run	Post-run	Pre-run	Post-run	Pre-run	Post-run
Group	3.01 ± 0.43	3.36 ± 0.44	20.30 ± 3.80	21.98 ± 4.15	152.4 ± 17.7	142.2 ± 14.0
Alone	3.10 ± 0.52	3.49 ± 0.51	20.75 ± 4.12	22.83 ± 4.38	153.1 ± 20.7	144.3 ± 14.2

TMD: total mood disturbance; POMS: profile of mood states.
